# Réduction de la létalité du paludisme après la mise en œuvre d'un plan d'urgence pour l'amélioration de la prise en charge des cas dans le district sanitaire de Bittou, Burkina Faso

**DOI:** 10.48327/mtsi.v4i1.2024.495

**Published:** 2024-02-29

**Authors:** Thierry Damien Adamo OUÉDRAOGO, Ousmane BADOLO, Youssouf SAWADOGO

**Affiliations:** U.S. President's Malaria Initiative (PMI) Integrated Family Health Service Project

**Keywords:** Paludisme, Prise en charge, Taux de létalité, Bittou, Burkina Faso, Afrique subsaharienne, Malaria, Case management, Case-fatality rate, Bittou, Burkina Faso, Sub-Saharan Africa

## Abstract

**Objectif:**

Cette étude a pour objectif d'observer et de décrire la réduction de la létalité du paludisme chez les enfants de moins de 5 ans reçus dans le Centre médical avec antenne chirurgicale (CMA) de Bittou au Burkina Faso. Face à la létalité élevée du paludisme, l’équipe cadre du district a mis en place un plan d'urgence en 2016.

**Matériel et méthodes:**

Analyse des données collectées à partir des annuaires statistiques du ministère de la Santé du Burkina Faso de 2014 à 2021.

**Résultats:**

Le taux de létalité palustre des moins de 5 ans dans le district sanitaire de Bittou est passé de 1,39 % en 2014 et 1,52 % en 2015 à 0 % en 2016 et 2017, 0,2 % en 2018, 0 % en 2019, 0,07 % en 2020 et 0,05 % en 2021. La même tendance est observée au niveau du CMA avec 2,94 % et 2,59 % en 2014 et 2015, 0 % en 2016 et 2017, 0,38 % en 2018, 0 % en 2019, puis 0,17 % et 0,47 % en 2020 et 2021.

**Conclusion:**

La lutte contre le paludisme reste un défi au Burkina Faso. L'amélioration des taux de létalité observée à Bittou montre qu'une implication effective des équipes cadres des districts sanitaires peut contribuer à des réductions substantielles de la mortalité due au paludisme.

## Abstract (en)

**Objective:**

To observe the evolution in malaria case-fatality rate among children under 5 years of age receiving care at the Bittou district hospital (CMA) after an improvement of the care practices. The management team implemented an emergency plan in 2016 with 5 components: i) health facilities staff sensitization to enable rapid referral of severe malaria cases to CMA; ii) reorganization of CMA paediatric emergencies to make a physician as the mainpoint of contact; iii) ensuring availability of supplies for severe malaria case management, including the availability of blood; iv) daily medical check-ups of hospitalized patients; v) reinforcement of clinical staff skills at all peripheral health facilities. At the same time were introduced i) free care for children under 5 years; ii) municipality involvement to finance ambulance fuel for the referrals of patients; iii) free blood collection in professional schools and soldiers; iv) a free telephone line between the health structures; v) presence of 5 medical doctors at the CMA.

**Material and methods:**

Analysis of data collected from the statistical yearbooks of the Ministry of Health of Burkina Faso from 2014 to 2021.

**Results:**

The malaria case-fatality rate (CFR) in under-five in the Bittou health district (1.39% and 1.52% in 2014 and 2015) was higher than the average for all districts in this region (1.08%). After implementation of the emergency plan, the malaria CFR in Bittou declined to 0% in 2016 and 2017, 0.2% in 2018, 0% in 2019, 0.07% in 2020 and 0.05% in 2021. The same trend was observed at the CMA level with 2.94% and 2.59% in 2014 and 2015, 0% in 2016 and 2017, 0.38% in 2018, 0% in 2019, then 0.17% and 0.47% in 2020 and 2021.

**Conclusion:**

Malaria control remains a challenge in Burkina Faso. However, the improved malaria CFRs observed in Bittou show that effective involvement of health district teams could potentially contribute to substantial reductions in malaria case-fatality risk.

## Introduction

Le paludisme est un problème de santé publique majeur en Afrique subsaharienne. Au niveau mondial, l'Organisation mondiale de la Santé (OMS) a signalé une baisse de 18 % des cas de paludisme (de 262 millions en 2000 à 214 millions en 2015) et une baisse de 48 % des décès dus au paludisme (de 839 000 décès en 2000 à 438 000 en 2015). C'est en Afrique que cette baisse a été la plus prononcée (de 694 000 décès en 2000 à 292 000 en 2015). Cependant, le paludisme reste l'une des principales causes de mortalité infantile, en particulier en Afrique subsaharienne, tuant 1 enfant toutes les 2 minutes [10].

Au Burkina Faso, le paludisme était en 2015 la première cause de mortalité, de consultations externes (7 600 068 / 15 841 712 soit 48 %), d'hospitalisations (328 558 / 504 041 soit 65 %) et de décès (1 897 / 3 467 soit 52 %) dans les formations sanitaires de base, n'incluant pas les décès dans les structures de référence [5].

Le Burkina Faso comptait 1 698 formations sanitaires réparties dans les 13 régions sanitaires, dont 125 dans la région sanitaire du Centre-Est en 2015. Dans l'ensemble des formations sanitaires de la région, 784 567 cas de paludisme simple ont été enregistrés en 2015, dont 349 793 chez des enfants de moins de 5 ans avec 17 484 cas de paludisme grave et 282 décès soit un taux de létalité palustre de 1,61 % [5].

Durant la même période, 74 354 cas de paludisme simple ont été enregistrés dans le district sanitaire de Bittou, dont 33 701 chez des enfants de moins de 5 ans avec 1 249 cas de paludisme grave et 19 décès, soit un taux de létalité de 1,52 % [5]. Ce taux était supérieur à la moyenne des districts sanitaires du pays (5 379 décès parmi 450 042 cas de paludisme grave, soit un taux de létalité de 1,20 %). Le CMA de Bittou avait enregistré un taux de létalité de 2,94 *%* et de 2,59 % en 2014 et 2015.

Pour remédier à cette situation, l’équipe cadre de district (ECD) de Bittou a mis en place à partir de janvier 2016 un plan d'urgence pour la prise en charge des cas graves de paludisme arrivant au Centre médical avec antenne chirurgicale (CMA). Cette étude présente l’évolution du taux de létalité du paludisme chez les enfants de moins de 5 ans reçus dans le CMA de Bittou de 2016 à 2021.

## Méthodologie

### Présentation de la région du CentreEst et du district de Bittou

**Figure 1:**
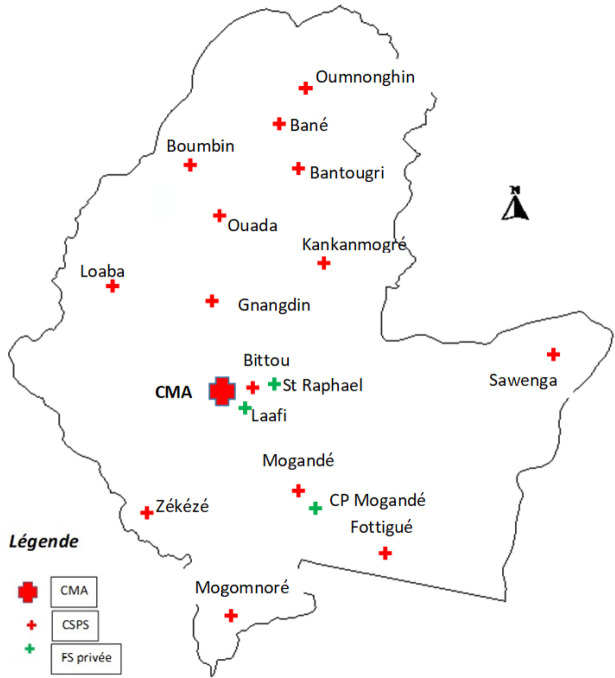
Carte sanitaire de la région du Centre-Est et du district sanitaire de Bittou (source : Carte sanitaire 2010 publiée en janvier 2012 par le ministère de la Santé) Map of the Centre-Est region and of the Bittou health district (source: 2010 health map published in January 2012 by the Ministry of Health

La situation du district sanitaire de Bittou dans la région du Centre-Est est présentée à la Figure 1.

CMA : Centre médical avec antenne chirurgicale; CP : Centre de santé privé; CSPS : Centre de santé et de promotion sociale; FS : Formation sanitaire

### Description des interventions

Le plan d'urgence établi en 2016 comprend les activités suivantes :

#### Dans les Centres de santé et de promotion sociale (CSPS)

- L'ECD a organisé des séances de sensibilisation ciblées pour les infirmiers chefs de poste (ICP) sur la nécessité de référer rapidement les cas graves de paludisme. Des messages téléphoniques leur sont régulièrement envoyés et un rappel est fait lors de chaque réunion entre l’équipe de santé et les ICP.
- Après la formation à la Prise en charge intégrée des maladies des enfants (PCIME), les ICP étaient sensibilisés à la reconnaissance des signes de danger du paludisme et au référencement des cas graves pour un traitement approprié dans les plus brefs délais.

#### Au CMA de Bittou

• Réorganisation des urgences pédiatriques. À l'arrivée, le patient est vu par le médecin de garde qui l'examine, lui administre un premier traitement et le surveille. La garde et la permanence sont assurées quotidiennement, à tour de rôle, par l’équipe des médecins et des infirmiers du CMA.

- Engagement du CMA à maintenir un stock suffisant de produits de santé, y compris de produits sanguins labiles, pour le traitement des cas graves de paludisme. La pharmacie et le laboratoire travaillent en collaboration avec le service médical d'urgence pour le maintien du stock.
- Examens médicaux quotidiens pour les patients hospitalisés permettant d'adapter les traitements.
- Renforcement des compétences du personnel soignant par des séances de formation hebdomadaires dirigées par un médecin.
- Suivi de proximité des cas de paludisme simple et grave et supervision trimestrielle du personnel des CSPS. L'ECD veille à ce que les CSPS soient en mesure de réagir rapidement aux signes de danger et qu'ils envisagent un transfert au CMA. Il s'assure également que les Comités de gestion des centres de santé (COGES) fournissent un soutien financier pour les dépenses en carburant.
- Communication sur le changement de comportement par le biais d’émissions de radio incitant les agents de santé à base communautaire (ASBC) à organiser le transfert des cas graves de paludisme des villages vers les CSPS.

D'autres améliorations du système de santé ont été introduites à la même période sans faire partie du plan d'urgence : i) la gratuité des soins pour les enfants de moins de 5 ans; ii) l'implication de la municipalité pour financer le carburant des ambulances pour le transfert des patients des formations sanitaires vers le CMA; iii) la collecte gratuite de sang dans les écoles professionnelles et au sein de l'armée; iv) l'existence d'une ligne téléphonique gratuite entre les structures; v) la présence de 5 médecins au CMA; et vi) le dynamisme et le coaching de l’équipe de gestion du district de santé.

### Type d’étude

Il s'agit d'une analyse des données du Système national d'information sanitaire (SNIS) sur la létalité du paludisme dans le district de Bittou, collectées à partir des annuaires statistiques du ministère de la Santé du Burkina Faso de 2014 à 2021.

### Collecte des données

Les données compilées dans les annuaires statistiques de 2014 à 2021 proviennent des rapports mensuels émanant des formations sanitaires, qu'elles soient publiques ou privées, et des centres de référence.

Le diagnostic biologique du paludisme repose sur deux techniques : l'examen microscopique à partir de gouttes épaisses et de frottis sanguins et/ou les tests de diagnostic rapide (TDR). Il complète le diagnostic clinique par le professionnel de santé.

Pour le traitement du paludisme simple, les médicaments recommandés au Burkina Faso sont les combinaisons fixes artésunate + amodiaquine, artémether + luméfantrine ou artésunate + pyronaridine. La quinine par voie orale est également recommandée. Les traitements en cas de forme grave sont l'artésunate injectable, l'artémether injectable et la quinine injectable avec un relais par la voie orale dès que possible.

Il est à noter qu'en mars 2016, le Conseil des ministres décidait la mise en œuvre des mesures de gratuité des soins au profit des femmes et des enfants de moins de 5 ans. Cette gratuité, qui couvrait à partir du 1^er^ avril 2016 trois régions (le Sahel, les Hauts Bassins et le Centre), s'est étendue à l’échelle nationale à partir du 1^er^ juin 2016. Cette initiative est venue accompagner les efforts de l'ECD de Bittou, débutés en janvier 2016, en renforçant la gratuité de la prise en charge du paludisme grave au CMA du Centre-Est.

### Traitement et analyse des données

Les prestataires de soins collectent les données dans un registre avant de les saisir dans le registre de données de santé (Endos-BF). Ces données (nombre de cas de paludisme confirmés par TDR ou/et goutte épaisse et décès dus au paludisme) ont été enregistrées dans les CSPS et le CMA. Les gestionnaires de données dans les districts et les régions sanitaires, puis à la Direction des statistiques et du système d'information sanitaire (DSSIS), effectuent des contrôles pour s'assurer de la qualité des données collectées.

Pour notre étude, les données collectées ont été :

- nombre de cas de paludisme simple chez les enfants de moins de 5 ans;
- nombre de cas de paludisme grave chez les enfants de moins de 5 ans;
- nombre de décès chez les enfants de moins de 5 ans;
- nombre de décès dus au paludisme chez les enfants de moins de 5 ans.

### Limites de l’étude

L’étude a utilisé des données sur les décès survenus dans les CSPS et le CMA du district sanitaire de Bittou. Le CMA est le centre de référence des CSPS. La majorité des décès et particulièrement ceux dus au paludisme grave y ont lieu. Nous ne disposons pas de données sur les enfants de moins de 5 ans décédés dans les villages de paludisme ou d'autres pathologies ou circonstances.

## Résultats

Les taux de létalité palustre chez les enfants de moins de 5 ans de 2014 à 2021 dans les districts sanitaires (DS) de la région sanitaire du Centre-Est sont présentés dans le Tableau I.

**Tableau I:** Taux de létalité moyen palustre chez les enfants de moins de 5 ans dans la région du Centre-Est de 2014 à 2021 Average malaria case-fatality rate among children under 5 in Centre-Est region, 2014-2021

**Centre-Est**	**2014**			**2015**			**2016**			**2017**		
	**Cas**	**DCD**	**Let%**	**Cas**	**DCD**	**Let%**	**Cas**	**DCD**	**Let%**	**Cas**	**DCD**	**Let%**
DS Bittou	935	13	1,39	1 249	19	1,52	1196	0	0,00	1 291	0	0,00
DS Garango	1 414	4	0,28	1 449	8	0,55	2497	10	0,40	2 925	15	0,51
DS Koupéla	3 950	86	2,18	3 604	81	2,25	2405	16	0,67	2 923	56	1,92
DS Ouargaye	4 448	64	1,44	3 480	45	1,29	4074	35	0,86	3 899	41	1,05
DS Pouytenga	1 941	9	0,46	2 226	12	0,54	2042	15	0,73	760	3	0,39
DS Tenkodogo	1 915	4	0,21	1 903	16	0,84	2175	2	0,09	2 439	1	0,04
DS Zabré	2 000	31	1,55	2 240	35	1,56	1700	30	1,76	2 090	8	0,38
**Taux de létalité moyen du Centre-Est**	**1,07**			**1,22**			**0,65**			**0,65**		
**Centre-Est**	**2018**			**2019**			**2020**			**2021**		
	Cas	DCD	Let%	Cas	DCD	Let%	Cas	DCD	Let%	Cas	DCD	Let%
DS Bittou	1 432	3	0,21	717	0	0,00	1 334	1	0,07	3 651	2	0,05
DS Garango	2 579	6	0,23	1 061	2	0,19	2 398	3	0,13	2 074	5	0,24
DS Koupéla	3 191	42	1,32	1 612	12	0,74	2 430	15	0,62	2 823	31	1,10
DS Ouargaye	3 084	29	0,94	2 156	28	1,30	4 060	70	1,72	4 453	25	0,56
DS Pouytenga	2 378	35	1,47	1 885	19	1,01	3 743	48	1,28	3 135	48	1,53
DS Tenkodogo	2 517	7	0,28	1 331	1	0,08	2 923	1	0,03	4 117	4	0,10
DS Zabré	3 122	15	0,48	1 174	9	0,77	2 484	20	0,81	1 993	37	1,86
**Taux de létalité moyen du Centre-Est**	**0,70**			**0,58**			**0,67**			**0,78**		

DS : District sanitaire; DCD : Décédés; Let : Létalité.

Dans la région du Centre-Est, les taux de létalité palustre moyen chez les enfants de moins de 5 ans étaient respectivement de 1,07 % et de 1,22 % en 2014 et 2015. De 2016 à 2021, les taux ont été inférieurs à 1 % variant de 0,58 % à 0,78 % (Tableau II).

**Tableau II:** Taux de létalité palustre chez les enfants de moins de 5 ans dans le district de Bittou de 2014 à 2021 Under-5 malaria case-fatality rate in Bittou health district, 2014-2021

DS BITTOU	2014	2015	2016	2017	2018	2019	2020	2021
Paludisme simple	37 582	33 701	16 703	48 937	45 546	23 174	43 500	41 106
Paludisme grave	935	1 249	1 196	1 291	1 432	717	1 334	3 651
Décès CSPS	0	3	0	0	0	0	0	0
Décès CMA	13	16	0	0	3	0	1	2
Total décès	13	19	0	0	3	0	1	2
Taux de létalité	1,39	1,52	0,00	0,00	0,21	0,00	0,07	0,05

CSPS : Centre de santé et de promotion sociale; CMA : Centre médical avec antenne chirurgicale.

De 2014 à 2015, le district sanitaire de Bittou avait chez les moins de 5 ans, un taux de létalité palustre dans ses formations sanitaires respectivement de 1,39 % et 1,52 %. De 2016 à 2019, le taux de létalité palustre est pratiquement nul, sauf en 2018. Ce taux remonte de 2020 à 2021 à un niveau faible (0,07 % et 0,05 %) (Tableau III).

**Tableau III:** Taux de létalité palustre chez les enfants de moins de 5 ans au CMA de Bittou de 2014 à 2021 Under-5 malaria case-fatality rate, 2014-2021, at Bittou CMA

CMA Bittou	2014	2015	2016	2017	2018	2019	2020	2021
Paludisme simple	2939	979	1571	2198	3468	1693	3246	1602
Paludisme grave	442	618	585	589	790	263	591	423
Décès paludisme	13	16	0	0	3	0	1	2
Taux de létalité	2,94	2,59	0,00	0,00	0,38	0,00	0,17	0,47

CMA : Centre médical avec antenne chirurgicale

Le taux de létalité au CMA de Bittou était de 2,94 % et de 2,59 % en 2014 et 2015. De 2016 à 2019, le taux de létalité est nul sauf en 2018 (0,38 %). De 2020 à 2021, une faible remontée est observée (0,17 % et 0,47 %).

## Discussion

La prise en charge des cas de paludisme simple se fait de plus en plus dans les formations sanitaires. Selon l'OMS, en 2015, 76 % des patients suspectés de paludisme en Afrique ont bénéficié d'un test biologique dont 74 % de TDR [9]. Cependant, malgré les efforts réalisés par ces formations sanitaires pour prendre en charge le paludisme grave, certains cas nécessitent un transfert rapide vers des centres de soins plus appropriés tels que les Centres médicaux (CM), les Centres médicaux avec antenne chirurgicale (CMA), les Centres hospitaliers régionaux (CHR) et les Centres hospitaliers universitaires (CHU). En général, les patients arrivent dans un état précaire en raison des retards dans la décision de transfert, de la distance entre le village et le centre de référence et de l'insuffisance des moyens techniques [3, 14]. La prise en charge du paludisme grave dans les centres de référence est fortement recommandée par l'OMS [7].

La prise en charge du paludisme grave est une préoccupation quasi quotidienne pour les praticiens travaillant dans les centres de référence. Ils reprochent souvent au niveau périphérique d'envoyer des patients moribonds. Les données de 2014 et 2015, avant la mise en place du plan d'urgence au CMA de Bittou, montraient un taux de létalité palustre particulièrement élevé à cause du retard de prise en charge de ces patients.

Un délai de plus de 24 heures avant le transfert dans un centre de soins est un facteur péjoratif [3]. À Brazzaville, Mabiala-Babela a rapporté que 238 cas avec un diagnostic de paludisme anémique grave ont été référés à l'hôpital après 5 jours. Ce délai augmente considérablement la mortalité hospitalière due au paludisme [4]. Selon Tamini, le taux de létalité était de 7,1 % chez les enfants de moins de 12 mois enregistrés au CHU Charles de Gaulle de Ouagadougou [13] après leur admission tardive.

T. H. Ramarokoto a rapporté des résultats similaires à Madagascar, indiquant que la part des décès liée au paludisme était de 7,2 *%* (n = 1267) de tous les décès signalés [12]. À l'hôpital gynéco-obstétrique et pédiatrique de Yaoundé, A. Chiabi a également rapporté un taux de létalité de 3,8 *%* pour le paludisme grave [2].

B. Camara *et al.* au Sénégal ont rapporté un taux de létalité palustre de 14,5 % dans les hôpitaux de Dakar [1] et N. C. Okoronkwo au Nigeria, un taux de létalité de 5,7 % avec 23,2 % attribués au paludisme sévère [6]. En Sierra Leone, A. Oxner a réduit la mortalité infantile due au paludisme grave de 9 % à 3,6 % en mettant en œuvre le protocole de traitement standardisé du paludisme de l'OMS [11]. Le district sanitaire de Bittou fait figure d'exception en organisant la prise en charge des cas de paludisme dans une vision de santé publique. La mise en œuvre des stratégies de lutte contre le paludisme et l'engagement des prestataires de santé périphériques et du CMA a contribué à éliminer les décès dus au paludisme à Bittou en 2016, 2017 et 2019.

Il est important de noter que la détermination, le charisme et la compassion de l'ECD de Bittou ont su insuffler un dynamisme pour l'atteinte de l'objectif, conduisant à ces résultats qui perdurent.

À partir de 2020-2021, la létalité palustre a modérément augmenté dans les districts sanitaires de la région du Centre-Est (de 0,7 % à 0,8 %) dont celui de Bittou (0,07 % - 0,05 %) et spécifiquement au CMA (0,17 % - 0,47 %). Ceci pourrait en partie s'expliquer par le renouvellement du personnel affecté dans d'autres localités, par la réduction des apports financiers au niveau des COGES et des mairies ainsi que par l'insécurité croissante dans la région du Centre-Est.

Au Burkina Faso, les cas de paludisme dans les villages sont un défi majeur en matière de santé publique. Souvent, ces cas ne sont pas systématiquement notifiés par les agents de santé à base communautaire (ASBC), en raison de divers obstacles tels que l'accessibilité géographique, le manque d'infrastructures adéquates, le manque de support de notification et parfois même des lacunes dans la sensibilisation des communautés locales. Les décès attribuables au paludisme, bien qu'ils puissent être significatifs, ne sont pas toujours formellement enregistrés, ce qui rend difficile l’évaluation précise de l'impact de la maladie au niveau communautaire, même si des efforts de collecte de ces données ont été faits à partir de 2017. Les notifications de cas de paludisme au niveau du CMA peuvent ne pas refléter pleinement la réalité du district dans son ensemble, car les données peuvent être biaisées en raison de la sousdéclaration. Les limites de représentativité des statistiques du CMA soulignent la nécessité d'une approche plus holistique et inclusive pour évaluer efficacement la prévalence et la gravité du paludisme dans l'ensemble du district de Bittou, afin de mettre en place des interventions ciblées et adaptées pour parvenir à zéro décès dû au paludisme.

Selon la stratégie technique de l'OMS pour la lutte contre le paludisme 2016-2030, l'objectif intermédiaire est de réduire la mortalité mondiale due au paludisme d'au moins 40 % d'ici 2020 par rapport à 2015 [8]. Pour atteindre cet objectif, chaque district sanitaire devrait travailler à la maîtrise de la létalité hospitalière. Il est possible de réduire drastiquement la létalité palustre dans un district sanitaire par des moyens simples et peu coûteux impliquant les COGES, la communauté, la collectivité et les prestataires de soins, accompagnés d'un programme continu de formation et de sensibilisation.

L'utilisation des nouvelles technologies, comme la formation continue des prestataires de soins à travers le mMentoring, le mHealth pour les agents communautaires dans les zones rurales reculées à Madagascar [12] et l'introduction des registres électroniques de consultation au Burkina Faso, sont autant de possibilités qui conduiront à une meilleure prise en charge du paludisme. Cependant, dans le contexte spécifique du Burkina Faso, au-delà de l'engagement financier des partenaires et de l’État, l'engagement des prestataires est l'atout le plus important pour la lutte contre la maladie dans les formations sanitaires.

## Conclusion

L'implication des acteurs de santé peut contribuer efficacement au contrôle de la morbidité et de la mortalité palustres. Le district sanitaire de Bittou, après la mise en œuvre d'un plan d'urgence, a maîtrisé le nombre de décès et n'a enregistré aucun décès dû au paludisme en 2016 et 2017. L'engagement de l’équipe cadre du district, à travers la mise en œuvre d'activités simples et complémentaires, a permis d'atteindre ces résultats. L'enthousiasme et la disponibilité de ces membres ont été déterminants pour obtenir des résultats célébrés par tous.

## Remerciements

Merci à Joanna WATSON pour la relecture de cet article.

## Contribution des auteurs

Thierry Damien Adamo OUEDRAOGO

- conception de l’étude;

- définition de la méthodologie;

- rédaction du manuscrit;

- prospection bibliographique.

Ousmane BADOLO

- rédaction, co-rédaction et validation du protocole;

- rédaction des procédures;

- réalisation de l’étude;

- relecture et validation du manuscrit.

Youssouf SAWADOGO

- analyse des données;

- validation des données;

- interprétation des résultats;

- réalisation de l’étude;

- relecture et validation du manuscrit.

## Liens d'intérêts

Les auteurs ne déclarent aucun lien d'intérêts.
